# Nuclear translocation of myocardin-related transcription factor-A during transforming growth factor beta–induced epithelial to mesenchymal transition of lens epithelial cells

**Published:** 2013-05-06

**Authors:** Madhuja Gupta, Anna Korol, Judith A. West-Mays

**Affiliations:** Department of Pathology and Molecular Medicine, McMaster University Health Science Centre, Rm 1R10, 1200 Main St. West. Hamilton, ON, L8N 3Z5, Canada

## Abstract

**Purpose:**

Transforming growth factor beta (TGFβ) is a known inducer of epithelial to mesenchymal transition (EMT), and studies in other systems have shown that nuclear localization of the myocardin-related transcription factor (MRTF) is downstream of TGFβ. In the following study, we investigated whether nuclear translocation of MRTF-A or MRTF-B is involved in TGFβ-induced EMT of lens epithelial cells (LECs). We further investigated the relationship between matrix metalloproteinase-2 and -9 (MMP-2/9) and MRTF in the EMT of LECs.

**Methods:**

Rat lens explant cultures were used as the model system. Explants were treated with TGFβ, an MMP-2/9 inhibitor, or actin binding drugs and immunostained for alpha smooth muscle actin (αSMA), MRTF-A, and MRTF-B. Cytoplasmic and nuclear intensities of cells were measured using ImageJ. Production of αSMA was measured using western blot analysis and ImageJ.

**Results:**

Untreated explant cells exhibited little αSMA expression, and MRTF-A and B were found to reside primarily in the cytosol. However, when stimulated with TGFβ, a significantly greater number of cells exhibited nuclear expression of MRTF-A, accompanied by an increase in αSMA expression. However, MRTF-B remained in the cytoplasm following TGFβ treatment. Cotreatment with an MMP-2/9 inhibitor and TGFβ resulted in reduced MRTF-A nuclear localization and αSMA expression compared to cells treated with TGFβ alone.

**Conclusions:**

Our results are the first to demonstrate the expression of MRTF-A in LECs and that its nuclear translocation can be stimulated by TGFβ. Our data further suggest that MMP-2 and -9 are involved in the translocation of MRTF-A in LECs during TGFβ-induced EMT.

## Introduction

Epithelial to mesenchymal transition (EMT) is involved in a wide range of biologic mechanisms, including wound healing responses and cancer metastasis [1-3]. EMT consists of a complex series of events, the hallmark of which is the loss of epithelial cell–cell and cell–basement membrane adhesion. Following loss of adhesion, cells transform into a mesenchymal- or myofibroblast-like phenotype and express contractile proteins such as alpha smooth muscle actin (αSMA) [3,4]. In the ocular lens, EMT is a feature of two pathologies or cataracts, anterior subcapsular cataract (ASC) and posterior capsular opacification (PCO) [5-9]. During ASC formation, which can occur following injury or with diseases such as atopic dermatitis, the lens epithelial cells (LECs) transform into spindle-shaped myofibroblast cells, which form fibrotic plaques beneath the lens capsule. These transformed myofibroblasts deposit an aberrant amount of extracellular matrix (ECM) such as type I collagen and express αSMA, both of which contribute to a loss in transparency of the lens. PCO, also known as secondary cataract, occurs as a post-cataract surgery complication and is reported in 20%–40% of patients within 2 to 5 years after surgery [10]. Following replacement of the cataractous lens by an artificial intraocular lens (IOL), any remaining LECs can migrate to the posterior aspect of the lens capsule, where they undergo EMT, deposit ECM, and cause capsular wrinkling, which results in a loss of transparency and disruption of vision despite IOL presence [11-13].

Various growth factors such as transforming growth factor beta (TGFβ), fibroblast growth factor, and epidermal growth factor trigger EMT in various cell systems. However, among these, TGFβ plays the most predominant role in the development of ASC and PCO. The presence of biologically active TGFβ has been reported in patients with ASC, and elevated levels of active TGFβ are present in the ocular media of patients undergoing cataract surgery [14,15]. In addition, in several cataract models, including whole rat lenses and rat lens explants, TGFβ induced LECs to undergo EMT-like changes, express increased amounts of αSMA, and form ASC plaques reminiscent of those observed in humans [6,16-18].

TGFβ operates through multiple signaling pathways, the most common of which involves the Smad proteins. Smad3 is a major mediator of TGFβ-induced fibrosis in the kidney and lung [19-21]. However, the role of Smad3 in the EMT of epithelial cells, and in particular, the EMT of LECs, is more complex [22] and remains controversial. In a lens injury model in mice that induces ASC, Smad3 signaling is activated upon injury, yet can be blocked by TGFβ neutralizing antibodies [23]. Furthermore, in mice lacking Smad3 (Smad3 knockout [KO] mice) ASC do not develop following lens injury, suggesting that the Smad3 pathway is required for this capsular fibrosis [24,25]. However, using two additional models, one of which employs an adenoviral TGFβ method, and the other a TGFβ-1 lens-specific transgenic mouse model, our laboratory has demonstrated that in the absence of Smad3, mice developed ASC plaques, which were immunoreactive to αSMA [9,26]. These data suggest that additional TGFβ-induced signaling cascades are involved in the EMT of LEC and ASC formation.

TGFβ-induced EMT also occurs through Rho GTPase-mediated actin dynamics. For example, reorganization of the cell cytoskeleton through actin polymerization involves changes in G (globular)-actin into F (filamentous)-actin, and this in turn can cause EMT [27]. Actin-binding proteins (ABPs) are responsible for relaying changes in the actin configuration of the cell to the nucleus. Recent evidence suggests that an important family of ABPs, the myocardin-related transcription factors (MRTFs), are important in regulating the EMT involved in the fibrosis of several tissues [28]. Within the cell, under resting conditions, the RPEL domains at the amino termini of MRTF form a stable complex with monomeric G-actin, resulting in the sequestration of MRTFs in the cytoplasm. However, following actin polymerization, in which G-actin is recruited into F-actin, MRTF dissociates from G-actin, and translocates to the nucleus where MRTF associates with serum response factor (SRF) and drives gene transcription of several muscle-type and cytoskeletal genes including αSMA known to be involved in EMT [29-33]. Two MRTFs have been identified in mammalian species, MRTF-A and MRTF-B [34], which are closely related to each other in structure and function [34,35]. Both transcription cofactors remain in the cytoplasm in untreated cells. However, upon TGFβ treatment, MRTF-A accumulates in the nucleus faster than MRTF-B [36]. MRTF-A is also more ubiquitously expressed than MRTF-B [35].

TGFβ-induced EMT events have been associated with increased expression of a family of zinc-dependent endopeptidases, the matrix metalloproteinases (MMPs), which are key regulators of tissue remodeling during embryonic development and wound healing. MMPs have also been implicated in several ocular diseases, including retinal disease, glaucoma, and corneal disorders [37], and more recently, evidence suggests MMPs play an integral part in the mechanism involved in ASC formation. More specifically, MMP-2 and MMP-9 play critical roles in this process. For example, previous studies from our laboratory demonstrated that MMP-2 and MMP-9 secretion was enhanced in the conditioned media of rat lenses following TGFβ treatment and subsequent ASC formation [38]. Furthermore, co-treatment of this in vitro lens cataract model with TGFβ and a specific MMP-2/9 inhibitor suppressed ASC plaques that were otherwise visible when the lenses were treated solely with TGFβ [38]. However, the precise role of these MMPs in EMT in the lens has yet to be determined.

To date, investigation of MRTF in the eye and specifically the lens has not been performed. Thus, in the current work we examined the localization of MRTF-A and MRTF-B in an ex vivo rat lens explant model following TGFβ stimulation and correlate this with the expression of αSMA, a known marker of the EMT. We further determine the requirement of nuclear translocation of MRTFs in the EMT of lens epithelial cells by manipulating MRTF localization using actin-binding drugs. Finally, we have examined the relationship of the matrix metalloproteinases, MMP-2/9, previously shown to play an important role in the EMT of LECs, in MRTF translocation.

## Methods

### In vivo rat lens epithelial explants

All animal studies were performed according to the Canadian Council on Animal Care Guidelines and the Association for Research in Vision and Ophthalmology (ARVO) Statement for the Use of Animals in Ophthalmic and Vision Research. Lenses were dissected from 17- to 19-day-old Wister rats (Charles River Laboratories, Montreal, Canada) after euthanizing them first with CO_2_ asphyxiation following cervical dislocation. Lenses were placed on 35 mm Petri dishes with the posterior side facing up in serum-free M199 media (Invitrogen, Burlington, Canada) with antibiotics [39,40]. Petri dishes were incubated previously at 37 °C with the media. An incision was made at the posterior suture of the lens, and the epithelium was gently peeled back on all sides. The epithelium was then pinned to the Petri dish with the epithelial cells facing up. The fiber mass of the lens was discarded [8]. In lens explant cultures, epithelial cells grow in a Petri dish while still attached to the basement membrane. After explanting, the culture medium was replaced with fresh M199. Explants were kept in a 37 °C incubator with 5% CO_2_ for at least 48 h before treatment. Care was taken to choose only fully confluent explants with an even honeycomb structure of a single layer of cells for further treatments.

### Cell culture and reagents

All the cell culture reagents were bought from Invitrogen. The medium for cell explanting consisted of M199 (Invitrogen) supplemented with 1% penicillin/streptomycin, 1% Fungizone (amphotericin B), and 0.1% gentamycin. Explants were further treated with 6 ng/ml human recombinant TGFβ 2 (R&D Systems Inc., Minneapolis, MN). Two types of actin-binding drugs were used. Cytochalasin D (CD; Calbiochem, Mississauga, Canada) was used at a concentration of 2 µM, and latrunculin B (LatB; Calbiochem) was used at a concentration of 0.3 µM. An MMP-2/9 inhibitor (MMP-2/9 inhibitor II, Calbiochem) was also used on explants at a concentration of 25 µM.

### Immunofluorescent staining and microscopy

The whole mount rat lens explants used for immunofluorescence were first fixed with 10% 'neutral buffered formalin (NBF) for 20 min. After fixation with NBF, the explants were detached from the Petri dish and transferred to a glass tube. The rest of the staining procedure was performed with the explants free-floating in the glass tubes. The samples were then incubated at room temperature with the permeabilizer (0.1% Triton-X 100 in 0.5% sodium dodecyl sulfate [SDS]) and 5% normal donkey or goat serum respectively for 1 h. For localization of alpha smooth muscle actin (αSMA) and MRTF-A, explants were incubated overnight at 4 °C with fluorescein isothiocyanate conjugated mouse monoclonal antibody (1:200; Cat# A3777, Sigma-Aldrich Ltd., Oakville, Canada), goat polyclonal anti-MRTF-A antibody (1:200; Cat# sc-21558, Santa Cruz Biotechnology Inc., Santa Cruz, CA), and rabbit polyclonal anti-MRTF-B antibody (1:200; Cat# sc-98989, Santa Cruz Biotechnology). Following incubation with the primary antibody, the explants were further incubated for 1.5 h at room temperature with Alexa Fluor 568 conjugate donkey anti-mouse secondary antibody (1:200; Invitrogen) for MRTF-A and Alexa Fluor 568 conjugate goat anti-rabbit secondary antibody (1:200; Invitrogen). To serve as a negative control, explants were treated with a non-specific, irrelevant primary antibody followed by the same secondary antibody. The explants were then mounted in ProLong Gold antifade mounting medium (Invitrogen) containing 4', 6-diaminodino-2-phenylindole (DAPI) as a nuclear stain. After the final wash with PBS, the explants were air dried thoroughly and coverslipped using ProLongGold antifade reagent (Invitrogen) as a mounting medium.

A Leica microscope was used to visualize the cells. Pictures were taken using Openlab software (Quorum Technologies Inc., Guelph, Canada). Resizing of the images and scale bars was performed using Photoshop (Adobe Systems Inc., San Jose, CA).

### Western blotting and quantification

Proteins were extracted from rat explant cultures. Explants were pooled in groups of four, and extracted proteins were examined using 10% SDS-polyacrylamide gel electrophoresis. The explants were lysed using Triton-X 100 lysis buffer supplemented with a protease inhibitor cocktail (Roche Canada, Mississauga, Canada). Cells were lysed using four pulses of 3 s each (with incubation on ice in-between) using a sonicator. Total protein concentration of the lysates was determined using Bradford protein assay (Bio-Rad Laboratories, Mississauga, Canada). Equal amounts of total proteins were loaded in 10% SDS polyacrylamide gels. The proteins were then electrotransferred onto a nitrocellulose membrane (Pall Corporations, Pensacola, FL). Membranes were blocked with Odyssey Blocking Buffer (LI-COR Biotechnology, Lincoln, NE) and incubated at room temperature for 1 h. Incubation with primary antibody was done at 4 °C with a combination of 1:8,000 mouse monoclonal anti-αSMA (Cat# A2547, Sigma-Aldrich Canada) and 1:10,000 chicken polyclonal anti-β III tubulin (Cat# ab41489, Abcam Inc., Toronto, Canada). Beta-tubulin was used as a loading control. Following the overnight incubation, corresponding LI-COR IRDye IR Dye secondary antibodies (800cw, 600cw) were used, and protein bands were visualized using an Odyssey Infrared Imager (LI-COR Biotechnology). Under the scanner, both bands fluoresced with different colors. The bands from the resulting pictures were then quantified using ImageJ software (software developed by Wayne Rasband, National Institutes of Health, Bethesda, MD). Protein expression was represented in bar graphs with standard error of the mean (±SEM) measurements.

### Image processing for quantification

Immunofluorescent pictures of different cellular treatments were analyzed using ImageJ software. Integrated intensities were measured from a rectangular area of fixed measurement. Measurements were taken from three random places from the cytoplasm and the nucleus of each cell. The ratios of the average nuclear and cytoplasmic intensities were calculated and placed into categories. Based on a previous study [28], to make these categories exact, the distribution data were verified using the nuclear/cytoplasmic ratios as <0.75 (cytosolic), 0.75–1.25 (even or pan-cellular), and >1.25 (nuclear). To quantify αSMA, total fluorescence of the images was normalized and measured. For each image, the total number of cells was determined, and the percent value of αSMA was measured and documented using ImageJ.

### Statistical analysis

For explant cultures, measurements were taken from 500 to 1,500 cells per treatment. A total of three to seven replicates were counted for each treatment. For western analysis, four explants were pooled for each set of data, and a total of four to six replicates were analyzed for each treatment. For αSMA quantification using ImageJ, a total of 300–1,000 cells were counted. Altogether, a total of three to six replicates were counted per treatment. All measurements were expressed as the mean±SEM and analyzed using the Student *t* test or one-way analysis of variance (ANOVA) (SPSS software, 19.0, Chicago, IL). A value of p<0.05 was considered statistically significant.

## Results

### Changes in localization of myocardin-related transcription factor-A and myocardin-related transcription factor-B in lens epithelial cells following treatment with transforming growth factor beta

**Figure 1:**
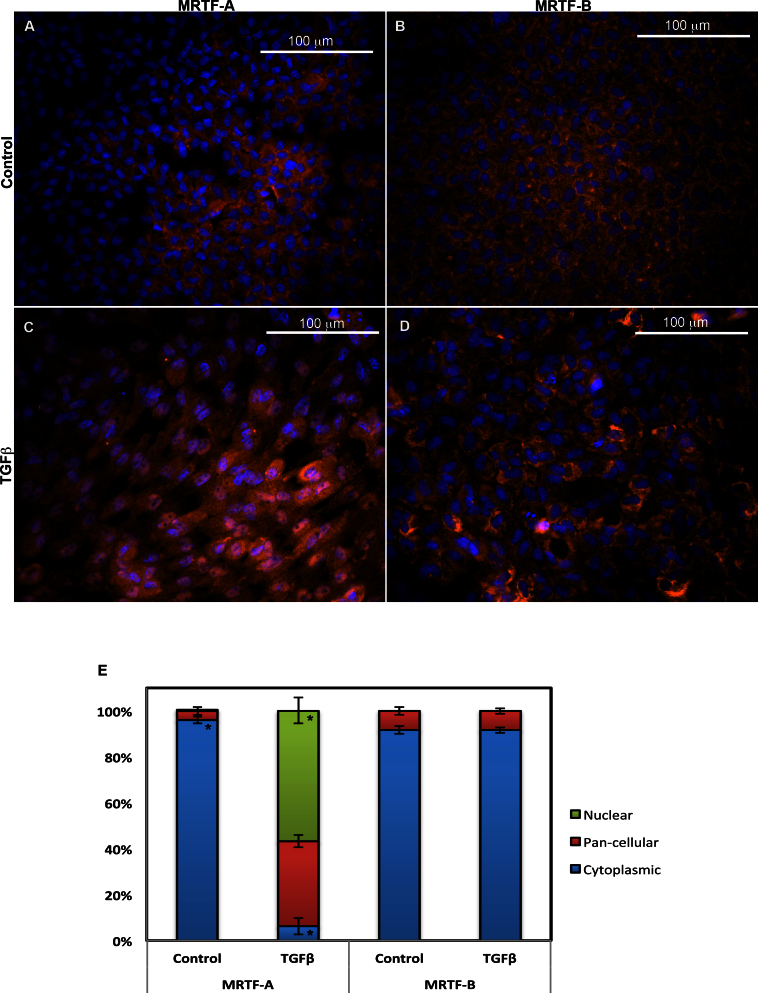
Localization and quantification of myocardin-related transcription factor-A (MRTF-A) and MRTF-B following transforming growth factor beta treatment. Cells were treated with 6 ng/ml transforming growth factor beta (TGFβ) for 48 h. The composite pictures shown here are stained with Alexa Fluor 568 conjugated secondary (red) for MRTF-A and -B and with 4', 6-diaminodino-2-phenylindole (DAPI) as a nuclear stain (blue). Untreated (control) rat lens explants exhibit cells with primarily cytoplasmic MRTF-A (**A**) and MRTF-B (**B**). Following TGFβ treatment, MRTF-A appears to be primarily in the nucleus (**C**). MRTF-B, however, remains cytoplasmic despite treatment with TGFβ (**D**). Each scale bar equals 100 μm. Quantification and comparison of intracellular translocation of MRTF-A and MRTF-B in the rat lens explant cell cultures upon TGFβ treatment were conducted using ImageJ (**E**). MRTF-A and MRTF-B were primarily cytoplasmic in the control (n=3). A significant increase *(p<0.05) in nuclear MRTF-A and a significant decrease *(p<0.05) in cytoplasmic MRTF-A were observed after TGFβ treatment (n=7). However, in the case of explants stained for MRTF-B, no significant change was observed in the intracellular localization following TGFβ treatment (n=3).

Transcription factors MRTF-A and B are expressed in several tissues and can be localized in different compartments of the cell, which affects MRTF signaling [29,35]. For example, MRTFs can be localized primarily in the cytoplasm (cytoplasmic), remain evenly distributed throughout the cell (pan-cellular), or be predominantly present in the nucleus (nuclear) [28]. In our initial experiments, we localized MRTF-A and -B in untreated rat lens explants cultures and in explants following treatment with TGFβ2 (6 ng/ml) for 48 h. In the untreated explant cells, MRTF-A and MRTF-B exhibited predominantly cytoplasmic localization, whereas some cells showed pan-cellular expression (Figure 1A,B). Following TGFβ treatment, the explant cells exhibited primarily nuclear MRTF-A staining (Figure 1C). However, in comparison, MRTF-B remained predominantly cytoplasmic (Figure 1D). Thus, treatment with TGFβ primarily stimulated nuclear translocation of MRTF-A, but not MRTF-B, in rat lens explants cultures.

To quantify the changes in compartmental localization of MRTF-A and -B, image processing software ImageJ was employed. The fluorescent intensity of MRTF-A and -B protein in the cytoplasm and nucleus of the cells was measured and compared to determine the number of cells with the different compartment locations. As shown in Figure 1E, in untreated explants the number of cells with cytoplasmic MRTF-A was 96%. Following TGFβ treatment for 48 h, this decreased significantly to 6.2% (p<0.05). Correspondingly, the number of cells with nuclear MRTF-A increased significantly (p<0.05) from 0.1% in untreated samples to 56.8% in those treated with TGFβ. There was also an increase of 33% in the number of cells with pan-cellular MRTF-A following TGFβ treatment. Quantification of MRTF-B localization further demonstrated that treated and untreated explant cells had primarily (approximately 91%) cytoplasmic MRTF-B localization with little or no nuclear localization (Figure 1E). Thus, no significant change in intracellular localization of MRTF-B was detected following TGFβ treatment of the explant cells. These results demonstrate that TGFβ treatment primarily facilitates nuclear migration of MRTF-A in lens epithelial cells.

### Positive association between nuclear myocardin-related transcription factor-A and alpha smooth muscle actin expression by cells

**Figure 2:**
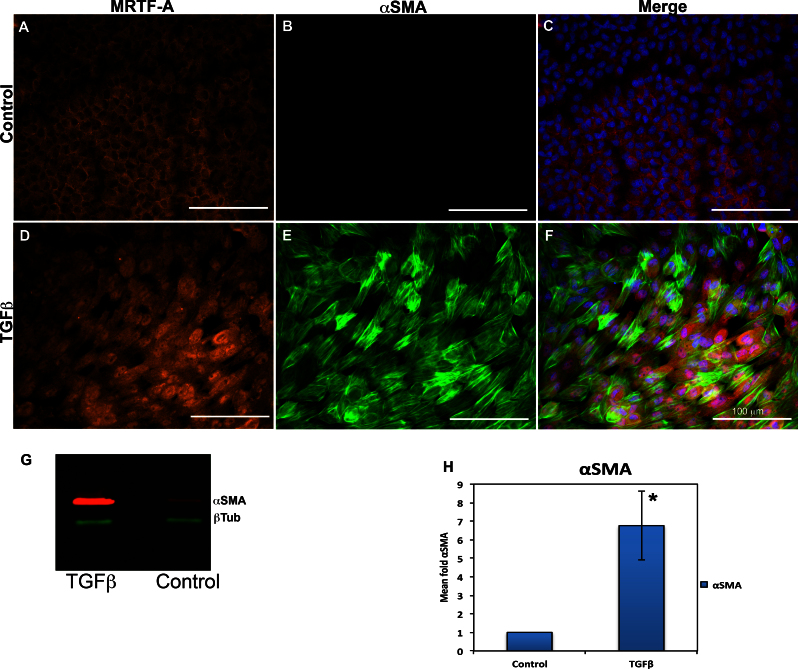
Rat lens explants treated with transforming growth factor beta (TGFβ) exhibit nuclear localization of myocardin-related transcription factor (MRTF-A). Cells were stained with an Alexa Fluor 568 conjugated MRTF-A secondary (red), fluorescein isothiocyanate for alpha smooth muscle actin (αSMA; green), and 4', 6-diaminodino-2-phenylindole (DAPI) for the nuclei (blue). The untreated (control) lens explants exhibited mostly cytoplasmic MRTF-A (**A**) and no αSMA expression (**B**). Explants treated with 6 µg/ml TGFβ for 48 h showed predominant nuclear localization of MRTF-A (**D**) along with robust αSMA expression (**E**). The corresponding composite picture with DAPI (**C**, **F**) demonstrates the difference between nuclear and cytoplasmic localization more effectively. Each scale bar equals 100 μm. Total protein was extracted from the rat lens culture cells and run on western gels. The western blot data clearly demonstrate an increase in αSMA expression (red) by the cells after TGFβ treatment (**G**). Levels of β-tubulin present was used as loading controls. Bands of αSMA were quantified with ImageJ (**H**). Results show a significant increase *(p<0.05) in αSMA production by the explants following TGFβ treatment (n=6) compared to the untreated controls (n=4).

MRTF translocation has also been linked to EMT in various tissues [28,36]. To further investigate this, rat lens epithelial explants treated with TGFβ and immunostained for MRTF-A and MRTF-B, as shown in Figure 1, were also stained for αSMA, a known mesenchymal marker used as an indicator of the EMT. These experiments revealed that untreated explants, which had predominantly cytoplasmic MRTF-A and MRTF-B localization (Figure 2A–C), exhibited little or no αSMA expression. In comparison, TGFβ-treated explants, which demonstrated primarily nuclear MRTF-A localization, showed substantial αSMA expression (Figure 2D-F). Interestingly, MRTF-B localization remained cytoplasmic despite substantial αSMA expression (not shown). Thus, MRTF-A, but not MRTF-B, nuclear translocation was associated with the EMT of the lens explant cells. Western blot analysis was performed to verify that TGFβ treatment resulted in a significant increase in αSMA production by explant cells (Figure 2G). When compared to the control, the resulting data clearly demonstrate a significant (fivefold, p<0.05) increase in αSMA by the explant cells (Figure 2H). Therefore, nuclear migration of MRTF-A is positively associated with EMT as measured by αSMA expression in LECs. As a result, in the subsequent experiments only MRTF-A localization was considered.

### Effect of actin binding drugs on myocardin-related transcription factor-A translocation and epithelial to mesenchymal transition

**Figure 3:**
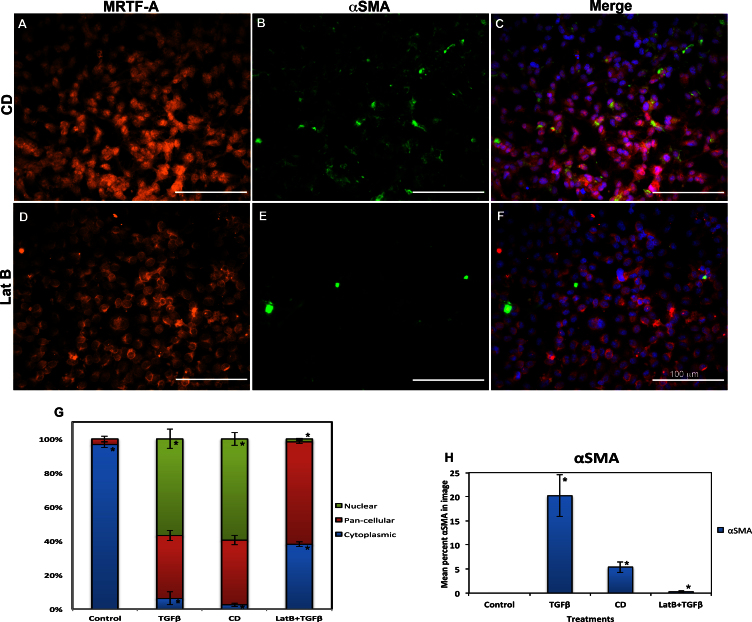
Effect of actin binding drugs on myocardin-related transcription factor (MRTF-A) migration in rat lens explants. Cells were immunostained with Alexa Fluor 568 conjugated MRTF-A secondary (red), fluorescein isothiocyanate for alpha smooth muscle actin (αSMA) (green), and 4', 6-diaminodino-2-phenylindole (DAPI) as a nuclear stain (blue). Treatment with cytochalasin D (CD) showed cells with bright nuclei as a result of increased nuclear accumulation of MRTF-A (**A**). In addition, there was an increase in αSMA production by the cells even in the absence of transforming growth factor beta (TGFβ). **B**: Latrunculin B (LatB)+ TGFβ cotreated cells showing mostly cytoplasmic and pan-cellular MRTF-A (**D**) with a decrease in αSMA expression (**E**). As a whole, the LatB+ TGFβ-treated cells greatly resemble the control cells. The corresponding composite picture with DAPI (**C**, **F**) demonstrates the difference between nuclear and cytoplasmic localization more effectively. Each given scale bar equals 100 μm. Quantification of MRTF-A localization in the rat lens epithelial explants was done using ImageJ (**G**). Compared to the control, the explant cells showed a significant increase in nuclear MRTF-A and significant decrease in cytoplasmic MRTF-A with TGFβ (n=7) and CD (n=4) treatment. However, when TGFβ treatment is compared to LatB and TGFβ cotreatment (n=4), the increase in cytoplasmic MRTF-A and decrease in nuclear MRTF-A are significant (*p<0.05). When the CD and TGFβ treatments are compared, the change in nuclear MRTF-A is non-significant. Similarly, the difference in nuclear MRTF-A between the control and LatB-TGFβ cotreatment is insignificant. Image analysis shows αSMA production by the cells (**H**). Results clearly demonstrate a significant increase *(p<0.05) in αSMA production on TGFβ (n=6) treatment along with a significant decrease *(p<0.05) in αSMA with LatB- TGFβ (n=4) cotreatment. In addition, there was also an increase in αSMA production with CD treatment (n=4).

Previous studies have shown that intracellular migration of MRTF-A is associated with actin cytoskeleton remodeling [29]. Therefore, two actin-binding drugs, CD and LatB, were used to determine if MRTF-A translocation could be manipulated in LECs. However, the drugs have different effects on the actin cytoskeleton. For example, LatB prevents the dissociation of the actin-MRTF complex, thus blocking nuclear accumulation of MRTF-A. On the other hand, CD interferes with the polymerization of actin molecules within the cell by influencing the G-actin to F-actin transition [41]. Thus, MRTF-A becomes liberated and migrates to the nucleus as G-actin is pulled away from the complex. Rat lens explants were treated first with CD (2 µM) alone (Figure 3A–C). Immunofluorescence of MRTF-A was performed and quantified using the image processing software ImageJ, as described previously. Results showed that following CD treatment there was a significant increase (59.4%) in the number of cells with nuclear localized MRTF-A (p<0.05; Figure 3 A–C,G). Furthermore, a corresponding, significant decrease (93.5%; p<0.05) in cytoplasmic MRTF-A was observed in the CD-treated explant cells, compared to untreated cells. Compared to the experiments shown in Figure 2, the CD-treated explants exhibited a similar number of cells with nuclear MRTF-A localization compared to explants treated with TGFβ (56.8%; Figure 3 A-C,G).

In the next set of experiments, the explants were cotreated with LatB (0.3 µM) and TGFβ (6 ng/ml). LatB binds with the MRTF-A-G-actin complex and prevents the detachment of G-actin from the complex. As a result, MRTF-A should be unable to dissociate from the complex and translocate to the nucleus, even in the presence of TGFβ. Explants cotreated with LatB and TGFβ clearly demonstrated significantly fewer cells with nuclear MRTF-A expression (1.7%), compared to those treated with TGFβ alone (56.8%; p<0.05; Figure 3D-G). Similarly, the cotreated explants exhibited a significantly higher number of cells with cytoplasmic (38.1%, p<0.05) and pan-cellular (60.3%) MRTF-A localization compared to explants treated with TGFβ alone (Figure 3D-G). MRTF-A was primarily cytoplasmic in the explants treated separately with LatB, and the cells did not show any fibrotic response (data not shown).

Immunographs of the explants treated with CD and LatB were assayed using ImageJ to quantify the number of cells expressing αSMA. This method was used due to the fewer number of cells available for western blot analysis. As seen in the immunograph (Figure 3), there is an increase in αSMA expression upon treatment with CD. ImageJ fluorescence assay also showed a 5% increase in αSMA when compared to control (Figure 3H). However, the increase was not comparable to that experienced with TGFβ treatment (>25%). When compared to TGFβ treatment alone, the LatB and TGFβ cotreated explants exhibited a significant decrease in αSMA expression (Figure 3E,H, p<0.05). Together, these findings demonstrated that manipulation of MRTF-A translocation with actin-binding drugs altered the translocation of MRTF-A and subsequent EMT of lens epithelial cells, as determined by αSMA expression.

### Effect of matrix metalloproteinase-2 and -9 inhibitors on myocardin-related transcription factor-A localization

**Figure 4:**
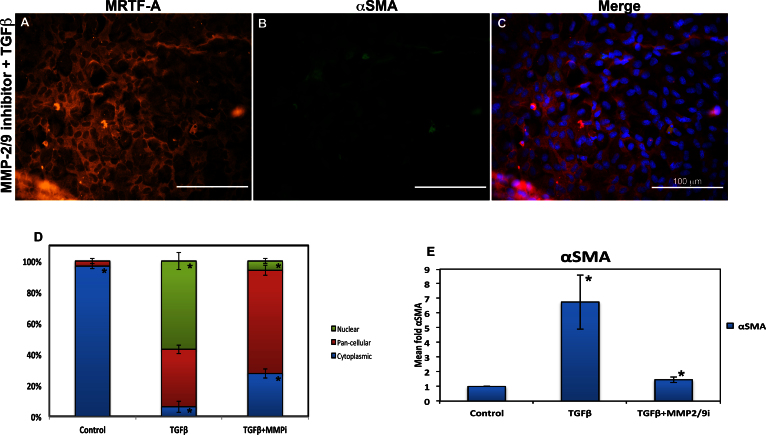
Effect of matrix metalloproteinase-2/9 (MMP-2/9) inhibitor on transforming growth factor beta (TGFβ)–induced myocardin-related transcription factor (MRTF-A) migration in rat lens explants. Rat lens explants were cotreated with TGFβ and MMP-2/9 inhibitor and immunostained with Alexa Fluor 488 conjugated MRTF-A secondary (red), fluorescein isothiocyanate (FITC) for alpha smooth muscle actin (αSMA; green), and 4', 6-diaminodino-2-phenylindole (DAPI) as a nuclear stain (blue). Cotreatment showed mostly cytoplasmic and pan-cellular MRTF-A (**A**) localization. There was also decreased αSMA expression in the cotreated cells (**B**) compared to those treated with TGFβ alone (see Figure 2). The composite image provides a clearer picture of the different intracellular localizations (**C**). Each scale bar given equals 100 μm. Quantification of intracellular translocation of MRTF-A was performed with ImageJ (**D**). Untreated (control) explants exhibited primarily cytoplasmic MRTF-A. Following treatment with TGFβ, a significant increase in nuclear MRTF-A localization was observed. When compared to TGFβ treatment alone, the TGFβ and MMP-2/9 inhibitor cotreated cells exhibited a significant decrease in cytoplasmic and nuclear MRTF-A (n=7, p<0.05). Production of αSMA was quantified with western blots and ImageJ (**E**). Quantification results demonstrate a corresponding significant increase *(p<0.05) in αSMA expression following TGFβ treatment (n=6) compared to untreated explants (n=4). A significant decrease *(p<0.05) in αSMA was also observed in TGFβ +MMP-2/9 inhibitor cotreated cells (n=4) compared to those treated with TGFβ alone.

Previous studies from our laboratory have shown that MMP-2 and -9 are involved in TGFβ-induced EMT in the lens. For example, we have shown that in whole rat lenses, the MMP-2/9 inhibitor (MMPi) can suppress TGFβ-induced EMT in the lens (as determined by αSMA expression) and subsequent subcapsular cataract formation [38]. To determine the involvement of MMP-2 and -9 in TGFβ-induced MRTF-A translocation, rat lens explant cultures were treated with TGFβ or cotreated with TGFβ, and 25 µM of the MMP-2/9 inhibitor (MMPi) for 48 h and subsequently stained for MRTF-A and αSMA. These studies revealed that compared to the TGFβ-treated explants those cotreated with TGFβ and the MMPi exhibited significantly fewer cells with MRTF-A nuclear localization (56.8% versus 6%, p<0.05; Figure 4A-D), and this corresponded with a reduction in αSMA expression (Figure 4E, p<0.05). A significant increase in cytoplasmic MRTF-A was also observed with the cotreatment (21.4%, p<0.05). Treatment of the explants with the MMPi alone did not result in any change in MRTF-A translocation or αSMA expression compared to the untreated explants (data not shown). Therefore, inhibiting MMP-2/9 resulted in reduced nuclear translocation of MRTF-A and subsequent αSMA expression in the LECs, suggesting that these MMPs may play an upstream role in regulating MRTF-A translocation.

## Discussion

The Rho/Rho kinase (ROCK) pathway is a TGFβ responsive signaling pathway, which involves reorganization of the cell cytoskeleton and is known to induce EMT via ABPs [27,42,43]. An important group of ABPs recently identified that have been shown to participate in EMT are the MRTFs [27,29,36,44]. In the current study, we sought to investigate the role of MRTFs in the EMT of LECs using a rat lens epithelial explant system. Our study is the first to demonstrate that MRTF-A and MRTF-B are expressed in LECs, but that only MRTF-A translocates to the nucleus in response to TGFβ and drug-induced changes in the actin cytoskeleton. We further demonstrate that shuttling of MRTF-A from the cytoplasm to the nucleus in LECs from all treatment groups was positively associated with expression of the EMT marker αSMA.

MRTF-A and -B are MRTF isoforms, which share homology with the founding MRTF family member myocardin. However, unlike myocardin, which is specifically expressed in the cardiovascular system, the MRTFs are expressed more ubiquitously, and found in multiple tissue and cell types [45]. Of the two MRTFs, MRTF-A has been shown previously to be most responsive to TGFβ and is the first to translocate to the nucleus [36]. In agreement with those findings, the results of the current study show that in rat lens explant cultures treatment with TGFβ resulted in the majority of the MRTF-A being localized to the nucleus, compared to the largely cytosolic expression observed in untreated cells. MRTF-B, in contrast, remained in the cytoplasm following treatment with TGFβ. Our studies also revealed that with nuclear localization of MRTF-A in LECs there was an accompanying increase in αSMA expression, suggesting that MRTF-A may be at least in part responsible for regulating its expression. Further evidence to support this comes from findings from experiments in which explants and cells were treated with LatB, an actin-binding drug that effectively acts as an inhibitor of MRTF-A translocation by sequestering the factor in the cytoplasm. After adding LatB to TGFβ-treated cells, we observed a substantial reduction in αSMA expression compared to cells treated with TGFβ alone. CD, another actin-binding drug, when used, had the opposite effect on the LECs and stimulated MRTF-A nuclear translocation. CD influences the G- to F-actin transition, liberating MRTF-A. Interestingly, the stimulation of MRTF-A translocation by CD alone resulted in an induction of αSMA expression. Indeed, LatB and CD may have had additional effects on the cell, beyond that related to the cytoskeleton and MRTF-A translocation. However, these data show an important association of MRTF-A translocation and EMT of LECs.

The transition of epithelial cells into myofibroblasts, rather than fibroblasts is thought to be a further progression than EMT and involves a myogenic program termed EMyT (epithelial-myofibroblast transition). This program involves the induction of ECM components such as the collagens and the de novo production of myofibroblast proteins such as αSMA [22]. Recent studies examining EMyT in kidney tubule cells have shown that TGFβ alone is not sufficient to induce MRTF-A translocation and EMyT [22,28,46]. An additional prerequisite in these cells was an injury to, or the absence of, intercellular contacts. For example, only when these cells were cultured in low calcium or subjected to a scratch wound were they able to be induced by TGFβ to express αSMA. This has been shown to occur via disruption of E-cadherin junctions, a key feature in sensitizing the cells for MRTF translocation by stimuli such as TGFβ. Thus, two hits are required for the progression to EMyT. In comparison, as we have demonstrated in the current study, in LEC explants, TGFβ alone can induce MRTF-A translocation and the appearance of myofibroblasts. Interestingly, in previous studies we have shown that whole lenses or lens epithelial explants with intact epithelial monolayers, when treated with TGFβ, display dissolution of the E-cadherin junctions and liberation of E-cadherin fragments into the culture medium [26,38]. Why the lens epithelial system is more permissive to EMT/EMyT than the kidney is not currently understood. However, determining any difference(s) in response to TGFβ between the cell systems will be important for further understanding the myogenic program in these tissues in which fibrosis occurs.

MMPs are a family of matrix-degrading enzymes shown to be involved in numerous ocular diseases [37,38]. Importantly, recent studies have suggested a strong link between MMP expression and cataract formation [47,48], and studies from our laboratory have shown that MMP-2/9 are specifically required for TGFβ-induced EMT of LECs and ASC formation [38,48-53]. In the current study, we explored the possible connection between MMP-2/9 and MRTF-A in the EMT of LECs by cotreating cells with TGFβ and an MMP-2/9 inhibitor. Our findings demonstrated that the MMP-2/9 inhibitor substantially reduced nuclear localization of MRTF-A induced by TGFβ, although not back to the low levels seen in untreated cells. Similarly, the αSMA expression levels were significantly suppressed following cotreatment with the MMP inhibitor. Together, these data suggest that MMP-2/9 may act upstream of MRTF-A translocation to mediate αSMA expression.

The mechanism by which MMPs control MRTF translocation is not known. However, several studies have shown that MMPs promote EMT by altering the E-cadherin/β-catenin pathway [54-57]. In particular, the association between E-cadherin and β-catenin is vulnerable to enzymatic attack by multiple MMPs, including MMP-9 and MMP-2 [43,57-59]. In support of this hypothesis, in the lens we have also shown that during TGFβ-induced ASC formation, E-cadherin fragments are detected in the culture medium [38]. In addition, cotreatment with MMP inhibitors has stabilized E-cadherin junctions and suppress E-cadherin disruption/shedding [55-57]. Since MRTF translocation has been shown to occur following disruptions in E-cadherin junctions, this may be the mechanism by which MMPs mediate MRTF translocation. Additionally, a link between Rho-GTPase activation and MMP expression has been established in different cell types, furthering the hypothesis that Rho-dependent pathways are intertwined with TGFβ’s induction of MMPs and subsequent EMT [60,61]. Interestingly, MRTF-A localization is also linked to Rho-GTPase activation. Further studies are necessary to determine the precise relationship among MMPs, the activation of Rho, and MRTF-A.

In summary, this study is the first to demonstrate the localization of MRTF-A in LECs and further show it is responsive to TGFβ. We have also shown that MRTF-A localization is highly correlated with the EMT of LECs, as demonstrated by the significant changes in αSMA expression. MMP-2/9, known mediators of TGFβ-induced EMT in the lens, were also shown to manipulate MRTF-A translocation. Further studies are required to demonstrate the relationship between MMPs and MRTF translocation in the EMT of LECs. Nonetheless, these data suggest that targeting MMP-2/9 or MRTF-A may be promising avenues for preventing the EMT of LECs, a feature of the lens fibrotic disorder ASC, as well as secondary cataract (PCO).
